# Protection Against *Toxoplasma gondii* Lethal ME49 Challenge Induced by Influenza Virus-like Particles Containing Dense Granule Protein 14

**DOI:** 10.3390/pharmaceutics18010093

**Published:** 2026-01-10

**Authors:** Jie Mao, Hae-Ji Kang, Gi-Deok Eom, Su In Heo, Hynnu Nam, Ji-Hyun Lee, Ki-Ho Park, Mi Suk Lee, Sung Soo Kim, Fu-Shi Quan

**Affiliations:** 1 Department of Biomedical Science, Graduate School, Kyung Hee University, Seoul 02447, Republic of Korea; maojie@khu.ac.kr (J.M.); ekd3910@khu.ac.kr (G.-D.E.); hsi7200@khu.ac.kr (S.I.H.); rino621@khu.ac.kr (H.N.); 1506wlgus@khu.ac.kr (J.-H.L.); 2 Medical Research Center for Bioreaction to Reactive Oxygen Species and Biomedical Science Institute, Core Research Institute (CRI), Kyung Hee University, Seoul 02447, Republic of Korea; sgskim@khu.ac.kr; 3 Department of Microbiology, Dongguk University College of Medicine, Gyeongju 38066, Republic of Korea; haedi1202@dongguk.ac.kr; 4 Department of Biology, Kyung Hee University, Seoul 02447, Republic of Korea; 5 Department of Infectious Diseases, Kyung Hee University Hospital, Kyung Hee University School of Medicine, Seoul 02447, Republic of Korea; parkkiho@khu.ac.kr; 6 Division of Infectious Diseases, Department of Internal Medicine, Kyung Hee University College of Medicine, Seoul 02447, Republic of Korea; mslee7@khu.ac.kr; 7 Department of Medical Zoology, School of Medicine, Kyung Hee University, Seoul 02447, Republic of Korea

**Keywords:** *Toxoplasma gondii*, GRA14, vaccine, virus-like particles, protection

## Abstract

**Background/Objectives**: *Toxoplasma gondii* (*T. gondii*) dense granule antigen 14 (GRA14) is a parasitophorous vacuole membrane protein that plays a critical role in the development of chronic-stage cysts. However, its potential as a vaccine antigen and long-term immunity have not been evaluated using a virus-like particle (VLP) platform. **Methods:** influenza matrix protein (M1)-based VLPs displaying GRA14 were generated. Female BALB/c mice were intranasally immunized with the VLP vaccine and orally challenged with lethal ME49 cysts either 10 weeks or 32 weeks after prime vaccination for short-term and long-term immunity evaluation, respectively. **Results:** GRA14 VLP vaccination elicited higher levels of *T. gondii*-specific IgG, IgG1, and IgG2a antibody responses in sera compared to non-immunized controls. Upon challenge infection, elevated IgG- and IgA-secreting plasma cells, germinal center B cells, and memory B cells were observed, and CD4^+^, CD8^+^ T-cells, as well as both Th1 (IFN-γ) and Th2 (IL-4, IL-5) cytokines, were also increased. For the short-term immunity study, vaccinated mice exhibited suppressed cerebral inflammation, significantly reduced brain cyst burdens, maintained stable body weight, and achieved 100% survival. For the long-term study, GRA14 VLPs sustained elevated IgG and IgG1 levels as well as conferred partial yet significant protection, with lower cyst loads and 83% survival. **Conclusions:** GRA14 VLPs induce durable, balanced humoral and cellular immunity and provide both short-term and long-term protection against lethal chronic toxoplasmosis, supporting their potential as promising vaccine candidates.

## 1. Introduction

Toxoplasmosis is caused by the obligate intracellular protozoan *Toxoplasma gondii* (*T. gondii*), with an estimated one-third of the world’s population infected [1]. Prevalence is especially high in parts of Latin America and tropical Africa, where infection rates can exceed 80%, and an estimated 40 million people are infected in the United States, prompting the CDC to classify toxoplasmosis as a neglected parasitic infection requiring targeted public health attention [2]. Although conventional chemotherapeutic regimens are available, these treatments are limited by notable toxicity and an inability to eliminate the bradyzoite cysts that persist during chronic infection [3]. This highlights the need for safe and effective vaccines, which represent a critical component of global toxoplasmosis control strategies.

To date, the only commercially available vaccine against *T. gondii* is the live attenuated S48 strain used in sheep, while all human vaccine candidates remain in the preclinical stage [4]. Vaccine development for parasitic pathogens is particularly challenging due to their complex life cycles, making the identification of antigens essential for protective immunity a critical step. Among the numerous candidates, dense granule proteins (GRAs) have attracted considerable attention because they are secreted immediately after host–cell invasion and play pivotal roles in parasitophorous vacuole (PV) formation, host–cell modulation, and the establishment of chronic infection [5]. Recent immunoinformatics analyses evaluating six GRA antigens have identified multiple B- and T-cell epitopes, revealing favorable immunogenicity, hydrophilicity, and non-allergenicity across candidates [6]. Consistent with these predictions, previous in vivo studies have shown that several GRA proteins not only participate in essential biological processes throughout the *T. gondii* lifecycle but also elicit protective humoral and cellular immune responses when incorporated into specific vaccine platforms [4,7,8,9,10,11].

Among the GRA family, GRA14 exhibits a distinctive transmembrane topology, being anchored to the PV membrane with its C-terminal domain exposed to the host cytosol and its N-terminal region oriented toward the vacuolar lumen [12]. This orientation not only provides favorable antigen exposure but also enables GRA14 to modulate PV membrane organization and mediate molecular trafficking, suggesting its essential role in parasite survival and host interaction [12]. Consistent with these functional attributes, GRA14 deletion has been shown to increase parasite virulence and elicit stronger immune activation, thereby restricting parasite expansion [13]. Building on this biological and immunological relevance, GRA14-targeting DNA vaccination has been shown to elicit parasite-specific immune responses, extend survival following RH strain challenge, and reduce parasite burden after a three-dose intramuscular regimen [14]. However, the level of protection remained incomplete, indicating that the immunogenicity of GRA14 requires further enhancement through more efficient antigen presentation and delivery strategies.

Virus-like particles (VLPs) offer a promising solution. They structurally mimic native virions while lacking genetic material, thereby providing an excellent safety profile [15]. Their highly ordered, multivalent antigen display and particulate architecture facilitate efficient B-cell receptor crosslinking, rapid uptake by antigen-presenting cells, and robust induction of immunity [16]. The successful application of VLP technology in parasitic vaccinology, exemplified by the world’s only licensed human malaria vaccine, further underscores the platform’s safety, efficacy, and translational potential [17]. Notably, influenza matrix protein 1 (M1)-based VLPs represent a particularly versatile platform, as M1 possesses strong intrinsic self-assembly capacity and assembles at the inner leaflet of host-derived membranes, enabling efficient incorporation and stable presentation of homologous as well as heterologous membrane-associated antigens [18]. Consistent with these advantages, our previous work has shown that *T. gondii* antigens can be effectively displayed on influenza-based VLPs, which elicit strong humoral or cellular immune responses and confer significant protection against lethal infection in mice [19,20,21]. However, GRA14 has not yet been incorporated into a VLP platform, and its protective efficacy, durability of immune responses, and underlying immunological mechanisms remain unexplored.

Based on these considerations, our present study aimed to construct a GRA14-presenting VLP using the influenza matrix protein M1 as the self-assembly core and to systematically evaluate its immunogenicity and protective efficacy. Using both short-term and long-term lethal ME49 challenge models, we assessed the humoral and cellular immune responses induced by GRA14-VLP vaccination and examined its effects on parasite burden and overall protection. This work provides experimental evidence supporting GRA14 as a promising vaccine antigen and offers an innovative VLP-based strategy for advancing *T. gondii* vaccine development.

## 2. Materials and Methods

### 2.1. Ethics Statement

All animal procedures were approved by the Institutional Animal Care and Use Committee of Kyung Hee University (Approval No., KHSASP-24–487). Animals were housed under SPF conditions with controlled temperature (22 ± 2 °C), humidity (50 ± 10%), and a 12-h light/dark cycle. Sterile chow and autoclaved water were provided ad libitum. Animals exhibiting signs of severe distress or reaching humane endpoint predefined as a loss of body weight exceeding 20% of the initial baseline were euthanized by CO_2_ inhalation followed by cervical dislocation.

### 2.2. Recombinant Plasmid Construction

The full-length amino acid sequence of *T. gondii* GRA14 was analyzed using the Phobius online server (http://phobius.sbc.su.se accessed on 3 July 2025). Total RNA was extracted from purified RH tachyzoites using the RNeasy Mini Kit (Qiagen, Redwood City, CA, USA), and first-strand cDNA was synthesized with the PrimeScript RT Reagent Kit (Takara Bio, Shiga, Japan) following the manufacturer’s protocol. The coding sequence of GRA14 (GenBank accession no. MH213492.1; 1227 bp) was retrieved from the NCBI GenBank database (website) and was amplified by reverse transcription PCR using primers containing EcoRI and KpnI restriction sites: forward, 5′-AAAGAATTCATGCAGGCGATAG-3′; reverse, 5′-TTA GGTACCCTATTCGCTTGGTCT-3′. The amplified PCR product was ligated into the pFastBac1 vector (Invitrogen, Waltham, MA, USA) pre-digested with the EcoRI/KpnI and then transformed into *E. coli* DH5α competent cells (Thermo Fisher Scientific, Waltham, MA, USA). Positive colonies were identified through blue–white screening on LB plates supplemented with X-gal and IPTG. Recombinant clones carrying the correct insert were verified by Sanger sequencing (Macrogen, Seoul, Republic of Korea).

### 2.3. Bacmid Generation and Baculovirus Production

The verified pFastBac1-GRA14 plasmid was transformed into *E. coli* DH10Bac (Invitrogen). Recombinant colonies were selected and confirmed by colony PCR using M13 universal primers: forward, 5′-GTTTTCCCAGTCACGAC-3′; reverse, 5′-CAGGAAACAGCTATGA-3′. Recombinant bacmid DNA was extracted using the Plasmid SV Mini Kit (GeneAll Biotechnology, Seoul, Republic of Korea) and then transfected into Sf9 insect cells (CRL-1711l; ATCC, Manassas, VA, USA) using Cellfectin^®^ II Reagent (Invitrogen) in Sf-900 II SFM medium at 27 °C in a non-CO_2_ incubator. Primary baculovirus (P1) stocks were harvested after 4 days post-transfection, and recombinant baculovirus (rBV)-induced cytopathic effects were monitored using a microscope (Leica DMi8; Leica Microsystems, Wetzlar, Germany). Viral amplification was performed by infecting fresh Sf9 cultures at a multiplicity of infection (MOI) of approximately 0.1, generating P2 and P3 stocks for subsequent VLP production.

### 2.4. VLPs Production, Purification, and Characterization

Sf9 cells were co-infected with GRA14-rBV and influenza matrix protein M1-rBV at a ratio of 3:1 and a total MOI of 0.1. At 3–4 days post-infection (dpi), culture supernatants were collected and clarified by centrifugation at 4750 rpm for 30 min at 4 °C. The clarified supernatant was subjected to ultracentrifugation at 30,000 rpm for 30 min at 4 °C using an Optima™ XPN-80 ultracentrifuge equipped with an SW32Ti rotor (Beckman Coulter, Brea, CA, USA). For further purification, VLP crude pellets were layered onto a discontinuous sucrose gradient (15%, 30%, and 60%) and ultracentrifuged at 30,000 rpm for 1 h. Visible opalescent bands corresponding to VLPs were collected, diluted in PBS, and washed by centrifugation under the same conditions. Final VLP preparations were resuspended in PBS and stored at −80 °C until use [22].

VLP characterization was performed as previously described [20]. Total protein concentrations were determined using the Pierce™ BCA Protein Assay Kit (Thermo Fisher Scientific). VLP morphology and structural integrity were examined by a JEM-1400Plus transmission electron microscopy (TEM; JEOL Ltd., Tokyo, Japan) operated at 120 kV. The presence of GRA14 and M1 proteins within VLP preparations was confirmed by Western blot analysis. Membranes were probed with mouse anti-*T. gondii*-positive serum (1:500) to detect GRA14 and with monoclonal anti-M1 antibody (1:2000; Invitrogen) to detect M1. Images were captured using a ChemiDoc™ XRS^+^ imaging system (Bio-Rad, Hercules, CA, USA), and molecular weights were estimated using Image J software (version 1.54g). Antigenicity of VLPs was assessed by ELISA, in which GRA14 VLP-coated plates were incubated with *T. gondii*-positive mouse serum as the primary antibody, whereas M1-only VLP-coated plates served as the negative control.

### 2.5. Animal Immunization and Challenge Infection

Thirty-six seven-week-old female BALB/c mice (17–18 g) were purchased from NARA Biotech (Seoul, Republic of Korea). Following one week of acclimation, mice were randomly assigned to three experimental groups (*n* = 12 per group): naive, naive + challenge (Cha), and GRA14 immunization. Each individual mouse was considered an experimental unit. The total sample size was determined based on prior literature involving preclinical vaccine studies. Mice in the GRA14 group received intranasal administration of 50 µg GRA14 VLPs formulated in 50 µL PBS at 4-week intervals for a total of three doses. Serum samples were collected 10 weeks after the prime immunization (wpv) to assess systemic antibody responses. At 12 wpv (0 dpi), mice were orally challenged with purified ME49 tissue cysts (short-term immunity; *n* = 8 per group). Cysts were obtained from the brains of chronically infected donor mice. At 35 dpi, four mice from each group were euthanized for the collection of spleens, bone marrow, and brain tissue to evaluate immunological parameters, inflammatory cytokines, and parasite burden. The changes in body weight and survival were monitored every five days starting from the day of challenge infection, and observations continued until all animals in the naive + challenge group reached humane endpoints. Naive mice received PBS only and were not subjected to infection, whereas naive + challenge mice received PBS immunization followed by ME49 cyst challenge.

To evaluate long-term immunity, remaining immunized mice (*n* = 4 per group) were then orally challenged with a lethal dose of ME49 cysts at 32 wpv. Serum samples were obtained at the same time to measure persisting antibody responses, and body weight and survival were monitored from the day of challenge infection. At 35 dpi, brain tissue was collected to quantify chronic parasite burden.

All animals were maintained under uniform husbandry conditions to reduce environmental fluctuations. Vaccinations, viral challenges, and subsequent sample collections for all experimental groups were carried out in parallel, and the sequence of treatment administration was alternated to mitigate operator-related bias. Although the spatial arrangement of cages and specific orders were not systematically randomized, all procedures followed standardized protocols to minimize potential confounding influences. No predefined inclusion or exclusion criteria were established prior to the study. Every enrolled mouse completed the entire immunization and challenge regimen, and no subjects or data points were removed during analysis. All specimens collected throughout the experiment fulfilled the required quality control benchmarks and were incorporated into the final dataset.

### 2.6. ELISA for Antibody, Antibody-Secreting Cell (ASC), and Cytokine Response Evaluation

For antibody response determination, ninety-six-well plates were coated overnight at 4 °C with *T. gondii* lysate antigen (TLA) at a concentration of 2 µg/mL in 0.05 M carbonate–bicarbonate buffer [23]. Plates were washed and blocked with 1% BSA for 2 h at 37 °C. Serially diluted serum samples from mice collected at 10 or 32 wpv were added and incubated for 1 h at 37 °C. Antigen-specific IgG, IgG1, and IgG2a were detected using HRP-conjugated goat anti-mouse antibodies (1:2000; Southern Biotech, Birmingham, AL, USA) for 1 h. Color development was performed using O-phenylenediamine dihydrochloride dissolved in citrate buffer containing 30% H_2_O_2_, and reactions were terminated with 2 N H_2_SO_4_. Absorbance was measured at 490 nm (A_490nm_).

For the assessment of ASC responses, splenocytes and bone marrow cells were isolated from mice at 35 dpi. Single-cell suspensions were prepared and cultured in RPMI-1640 complete medium supplemented with 10% FBS and stimulated with 4 µg/mL TLA for 5 days at 37 °C [24]. Following incubation, culture supernatants containing antibodies actively secreted by ASCs were collected. Levels of secreted IgG and IgA were quantified using HRP-conjugated goat anti-mouse IgG or IgA secondary antibodies and detected by ELISA procedures. Absorbance was measured at 490 nm.

For cytokine evaluation, splenocyte cultures were used to assess systemic immune responses, whereas brain tissue homogenates were analyzed to quantify local inflammatory responses associated with chronic *T. gondii* infection. Splenocytes were stimulated with TLA (4 µg/mL) for 5 days, and culture supernatants were collected. Brain tissues were homogenized in PBS, centrifuged at 6000 rpm for 10 min at 4 °C, and the resulting supernatants were diluted five-fold in assay buffer. Concentrations of IFN-γ, IL-4, and IL-5 in splenocyte supernatants, as well as IFN-γ and IL-6 in brain homogenates, were quantified using corresponding OptEIA™ ELISA kits (BD Biosciences, San Jose, CA, USA) according to the manufacturer’s instructions. Tetramethylbenzidine substrate was used for colorimetric detection, and reactions were stopped with 2 N H_2_SO_4_. Absorbance was recorded at 592 nm.

### 2.7. Flow Cytometry for Parasite-Specific Immune Subset Profiling

Splenocytes were isolated and prepared as single-cell suspensions, followed by stimulation with 4 µg/mL TLA for 5 h at 37 °C to induce antigen-specific activation signals [25]. After stimulation, Fc receptors were blocked with anti-mouse CD16/CD32 antibody for 15 min. Cells were subsequently stained with fluorochrome-conjugated monoclonal antibodies to identify specific immune cell subsets, including germinal center B cells (GL7-PE, B220-FITC), memory B cells (CD19-PE-Cy7, CD38-FITC, IgD-PE), and T-cell subsets (CD3-FITC, CD4-PE-Cy7, CD8-PerCP), for 30 min at 4 °C. All antibodies were purchased from BD Biosciences and used in accordance with the manufacturer’s instructions. After staining and washing, samples were acquired on an Accuri C6 flow cytometer (BD Biosciences), and data were analyzed using C6 Accuri software (version 1.0.264.21).

### 2.8. Brain Cyst Quantification

Brain tissues were homogenized in PBS and purified in a 45% Percoll gradient at 12,500 rpm for 30 min at 4 °C to enrich *T. gondii* cysts. After centrifugation, the cyst-containing fraction was collected, washed, and resuspended in PBS. Purified tissue cysts were then enumerated under a microscope using aliquots of the final suspension [26].

### 2.9. Statistical Analysis

Statistical analyses were performed using GraphPad Prism version 9.0 (GraphPad Software, San Diego, CA, USA). Data is presented as mean ± SD. The Shapiro–Wilk test was used to assess the normality of data distribution. Group comparisons were conducted using ANOVA, followed by Bonferroni’s post hoc test, or the Kruskal–Wallis test was applied, followed by Dunn’s multiple comparisons test. A *p*-value of < 0.05 was considered statistically significant.

## 3. Results

### 3.1. Construction, Validation, and Antigenic Characterization of GRA14 VLPs

**Figure 1:**
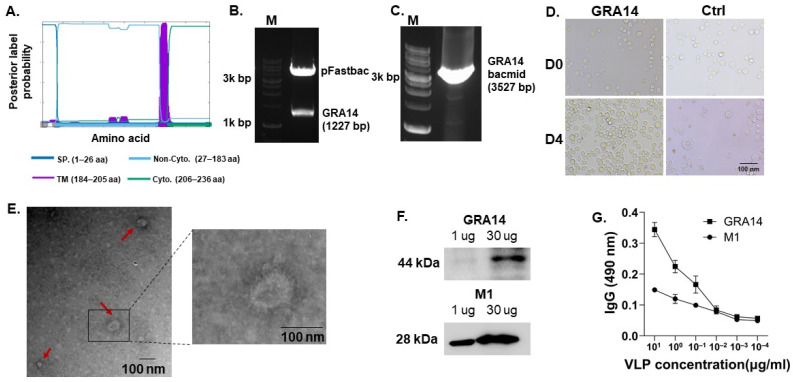
Characterization of VLPs. (**A**) Topological domain prediction of the full-length GRA14 amino acid sequence using the Phobius web server. (**B**) Agarose gel electrophoresis of the pFastBac1-GRA14 plasmid digested with EcoRI and KpnI to confirm successful cloning. (**C**) Colony PCR detection of the GRA14 gene in recombinant bacmid DNA extracted from *E. coli* DH10Bac transformants. (**D**) Morphological changes in sf9 cells transfected with GRA14-bacmid DNA. Cytopathic effects were observed at 96 h post-transfection. (**E**) Images captured by transmission electron microscopy of purified GRA14 VLPs revealed spherical particles with spike-like surface protrusions on the M1 matrix scaffold. The red arrows indicate VLPs. (**F**) Western blot analysis of VLPs by probing the membranes with polyclonal mouse anti-*T. gondii* sera and anti-M1 monoclonal antibody. (**G**) Serological reactivity of GRA14 VLPs was evaluated by ELISA using sera from *T. gondii* ME49-infected mice. Purified GRA14 VLPs were used as the coating antigen at an initial concentration of 10 µg/mL, followed by a 10-fold serial dilution series. Purified M1-only VLPs, normalized to the same starting protein concentration, were included as the negative control. *n* = 2 per group, with each sample measured in duplicate. Statistical analysis was performed using the Kruskal–Wallis test, followed by Dunn’s multiple comparisons test. VLPs, virus-like particles; SP, signal peptide; TM, transmembrane; Cyto., cytoplasm; M, marker.

Phobius analysis was performed to characterize the structural features of GRA14. The GRA14 protein was predicted to contain an N-terminal signal peptide (1–26 aa), followed by a non-cytoplasmic luminal region (27–183 aa), a single transmembrane helix (TM, 184–205 aa), and a short cytoplasmic tail (206–236 aa). These confirm that GRA14 is a membrane-associated protein that is structurally suitable for VLP surface display (Figure 1A). Successful cloning of the GRA14 gene (1227 bp) into the pFastBac1 vector (4775 bp) was validated by EcoRI/KpnI double digestion, producing two expected fragments corresponding to the vector backbone and the GRA14 insert (Figure 1B). The recombinant plasmid (pFastBac1-GRA14) was then introduced into DH10Bac *E. coli.* Colony PCR amplified a 3527 bp fragment, representing the GRA14-containing bacmid, thereby confirming successful recombinant bacmid generation (Figure 1C). Subsequent transfection of the GRA14 bacmid into Sf9 cells resulted in the production of GRA14-rBV. At 4 dpi, infected cells displayed typical baculovirus-induced cytopathic effects, including cell swelling and detachment, whereas control cells retained normal morphology (Figure 1D), indicating successful viral replication.

To assemble VLPs, Sf9 cells were co-infected with GRA14-rBV and M1-rBV. Transmission electron microscopy revealed spherical virus-like particles containing an M1 matrix core and peripheral surface protrusions consistent with GRA14 incorporation on the particle membrane (Figure 1E), demonstrating successful VLP formation. Protein expression was further confirmed by Western blotting, which detected a ~44 kDa band corresponding to GRA14 and a ~28 kDa band corresponding to M1 (Figure 1F), verifying expression of both structural components. Moreover, the antigenicity of GRA14 VLPs was evaluated by ELISA using *T. gondii*-positive mouse serum. Significant and concentration-dependent binding was observed when plates were coated with GRA14 VLPs, whereas M1-only VLPs exhibited minimal reactivity (Figure 1G). As such, these findings collectively demonstrate that GRA14 possesses the appropriate membrane topology for surface expression, that rBV and VLPs were successfully generated, and that the resulting GRA14 VLPs exhibit antigenicity with the potential to serve as a vaccine candidate.

### 3.2. GRA14 VLPs Induce T. gondii-Specific IgG and IgG Subtype Responses

**Figure 2:**
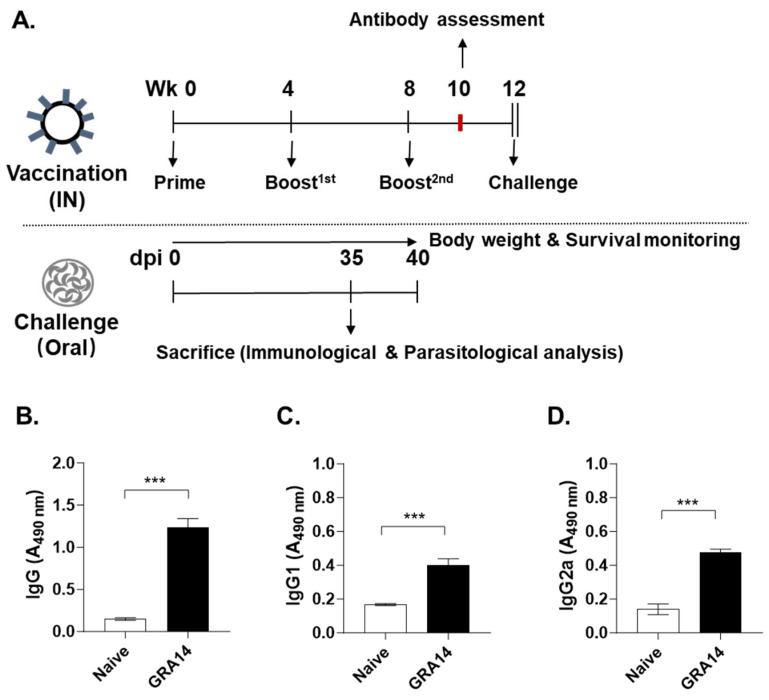
Antibody responses in the sera of mice. (**A**) Experimental schedule. BALB/c mice were intranasally immunized with GRA14 VLPs three times at 4-week intervals. At 12 wpv (0 dpi), the mice were orally infected with a lethal dose of *T. gondii* ME49 cysts. The mice were sacrificed to collect relevant tissues for further immunological parameters or parasitological determination at 35 dpi. Sera were collected at 10 wpv to assess (**B**) total IgG, (**C**) IgG1, and (**D**) IgG2a levels by ELISA, using *T. gondii* lysate antigens derived from ME49 cysts as the coating antigen. For each analysis, *n* = 4 per group, with each sample measured in triplicate. Statistical analysis was performed using ANOVA with Bonferroni’s post hoc test (GraphPad Prism 9.0). Data is shown as mean ± SD. *** *p* < 0.001. Wk, week; VLPs, virus-like particles; IN, intranasal; dpi, days post-infection.

Following VLP characterization, mice were intranasally immunized with GRA14 VLPs three times at 4-week intervals. Four weeks after the final boost, mice were orally challenged with a lethal dose of *T. gondii* ME49 cysts. Immune and parasitological parameters were evaluated using collected tissues at 35 dpi. Body weight and survival were monitored until 40 dpi (Figure 2A).

Serum samples were collected 10 wpv to quantify parasite-specific antibodies, whereas naive mouse serum served as the negative control. ELISA revealed that GRA14 VLP immunization induced a robust total IgG response, with titers increasing by approximately ten-fold compared with naive controls (Figure 2B). Analysis of IgG subclasses demonstrated significant increases in both IgG1 (Figure 2C) and IgG2a (Figure 2D) in the GRA14-immunized group relative to naive mice. Notably, the magnitude of increase for IgG1 and IgG2a was comparable, suggesting that GRA14 VLPs elicited a balanced Th1/Th2 immune profile. The antibody responses suggest that intranasal administration of GRA14 VLPs effectively induces significant and well-balanced humoral immunity, which is expected to contribute to protection against subsequent ME49 infection.

### 3.3. GRA14 VLPs Elicit Antibody-Secreting Cell (ASC) Responses in Spleen and Bone Marrow

**Figure 3:**
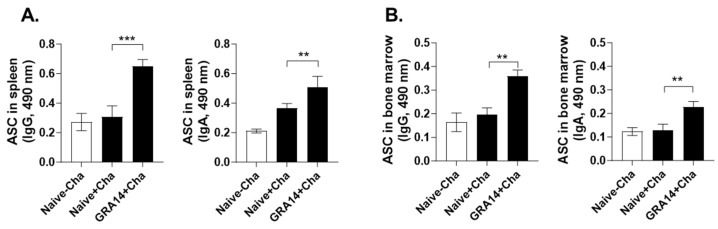
ASCs in the spleen and bone marrow. At 35 days post-infections, single cell suspensions of (**A**) spleens and (**B**) bone marrow were prepared and incubated with *T. gondii* ME49 lysate antigens for 5 days. Secreted IgG and IgA levels in culture supernatant were determined by ELISA. For each analysis, *n* = 4 per group, with each sample measured in triplicate. Statistical analysis was performed using ANOVA with Bonferroni’s post hoc test (GraphPad Prism 9.0). Data are presented as mean ± SD. ** *p* < 0.01, *** *p* < 0.001. ASCs, antibody-secreting cells.

To determine whether GRA14 VLP immunization induces long-lived humoral immunity, we isolated splenocytes and bone marrow cells at 35 dpi to evaluate ASC responses, which represent functionally active B cells that secrete antigen-specific antibodies. After 5-day incubation with *T. gondii* antigens, significantly higher levels of IgG and IgA were detected in the GRA14-immunized group compared with challenged controls in the spleen (Figure 3A). A similar trend was observed for ASCs in the bone marrow, with GRA14 VLPs markedly elevating IgG and IgA secretion relative to the control (Figure 3B). GRA14 VLP-induced ASC responses in secondary lymphoid tissues suggest effective generation of active and long-lived plasma cells that contribute to sustained antibody-mediated protection after ME49 challenge.

### 3.4. GRA14 VLPs Activate Germinal Center (GC) B Cells, Memory B Cells, and T-Cell Responses in Spleen

**Figure 4:**
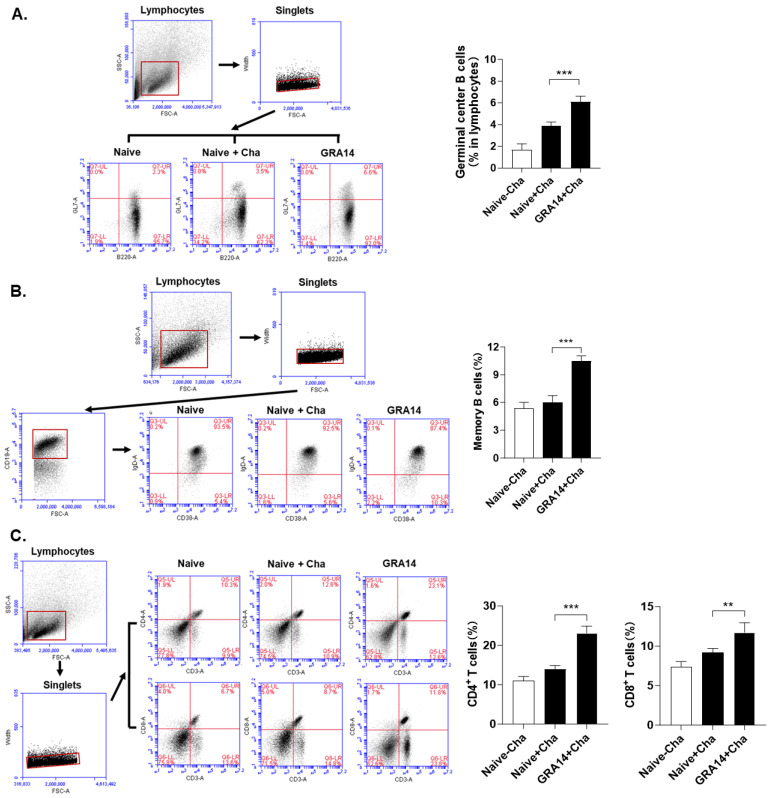
Immune cell responses in the spleen. At 35 days post-infection, splenocytes were collected from mice and subjected to ex vivo restimulation with ME49-derived lysate antigens. Frequencies of (**A**) GC B cells (GL7^+^B220^+^), (**B**) memory B cells (CD19^+^CD38^+^IgD^−^), (**C**) CD4^+^ and CD8^+^ T cells (CD3^+^CD4^+^ or CD3^+^CD8^+^) in the spleen were quantified in spleen lymphocyte populations by flow cytometry following staining by corresponding fluorescently labeled antibodies. Representative gating plots are shown, and the red numerical values displayed within each plot indicate the percentages of gated cell populations. For each analysis, *n* = 4 per group, with each sample measured in triplicate. Statistical analysis was performed using ANOVA with Bonferroni’s post hoc test (GraphPad Prism 9.0). Data is shown as mean ± SD. ** *p* < 0.01, *** *p* < 0.001. GC, germinal center.

At 35 dpi, splenocytes were collected, stimulated, and analyzed to determine the extent of adaptive immune responses. The frequency of GC B cells was significantly increased in GRA14-immunized mice compared with the naive challenge control (Figure 4A), indicating enhanced GC reactions and ongoing antibody refinement. Consistently, memory B-cell populations were also elevated in the GRA14 VLP group (Figure 4B), demonstrating efficient generation of long-lived humoral memory. Analysis of T-cell subsets showed substantial expansion of both CD4^+^ and CD8^+^ T-cell populations in GRA14-immunized mice relative to controls (Figure 4C). The rise in CD4^+^ T cells supports the observed enhancement of GC and memory B-cell responses, while the increased CD8^+^ T-cell population reflects a strengthened cytotoxic response critical for controlling intracellular ME49 infection. As such, GRA14 VLP may significantly augment splenic adaptive immunity by promoting robust GC B-cell activity, establishing strong memory B-cell pools, and enhancing both helper and cytotoxic T-cell responses.

### 3.5. GRA14 VLPs Enhance Th1- and Th2-Associated Cytokine Responses in Spleen

**Figure 5:**
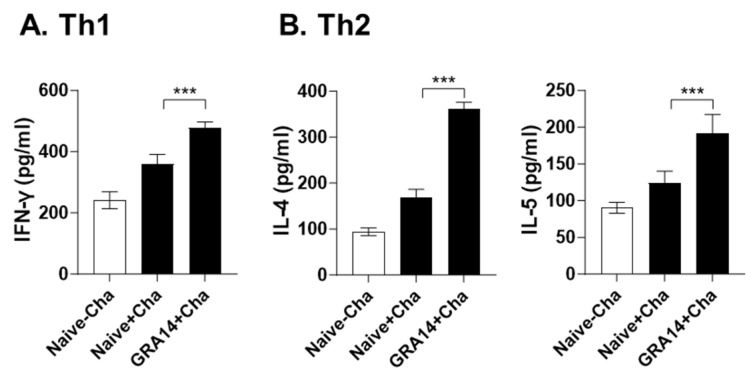
Immune cytokine concentrations in the spleen. Collected splenocytes were cultured with *T. gondii* ME49 lysate antigens for 5 days. Secreted cytokines, including (**A**) IFN-γ, (**B**) IL4, and IL-5 levels, were evaluated by ELISA. For each analysis, *n* = 4 per group, with each sample measured in triplicate. Statistical analysis was performed using ANOVA with Bonferroni’s post hoc test (GraphPad Prism 9.0). Data are presented as mean ± SD. *** *p* < 0.001.

Collected splenocytes were stimulated in vitro with *T. gondii* TLA to measure cytokine secretion. After 5-day stimulation, GRA14 VLP immunization induced a significant increase in IFN-γ production in culture supernatant compared with unimmunized groups (Figure 5A), indicating an increased Th1-type cellular response. The GRA14-immunized group also exhibited significantly higher levels of IL-4 and IL-5 relative to the control (Figure 5B), demonstrating that GRA14 VLPs effectively promote Th2-associated humoral activation. The combined elevation of these cytokines indicates that GRA14 VLPs induce a balanced Th1/Th2 cytokine profile, consistent with both effective B-cell activation and cellular immunity.

### 3.6. GRA14 VLPs Suppress Brain Inflammation, Cyst Formation, and Provide Protection Against Lethal ME49 Challenge

At 35 dpi, brain tissues were collected to determine inflammatory cytokines and parasite burden. IL-6 and IFN-γ levels were measured as indicators of local neuroinflammation, whereas cyst counts directly reflected the severity of chronic *T. gondii* infection in brain tissue. Body weight and survival were monitored to evaluate the overall protective efficacy.

**Figure 6:**
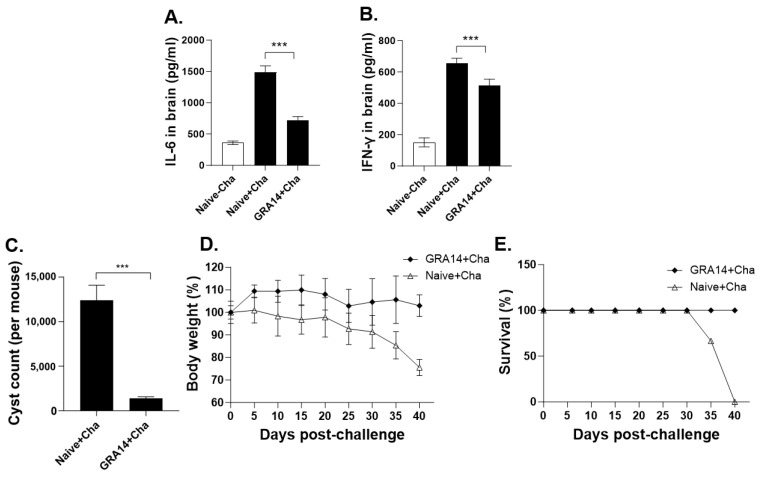
Cerebral inflammation suppression and protective efficacy. (**A**,**B**) Brain homogenates collected at 35 days post-infection were analyzed for pro-inflammatory cytokines IL-6 and IFN-γ by ELISA. (**C**) Quantification of cyst number in brain tissues under a microscope. (**D**) Changes in body weight and (**E**) survival rates were recorded at 5-day intervals for 40 days following challenge. For each analysis, *n* = 4 per group, with each sample measured in triplicate. Statistical analysis was performed using ANOVA with Bonferroni’s post hoc test (GraphPad Prism 9.0). Data are presented as mean ± SD. *** *p* < 0.001. For survival analysis expressed as the percentage of surviving animals per group at each time point, SD is not applicable.

GRA14-immunized mice exhibited a marked reduction in brain IL-6 levels compared with naive + challenge controls (Figure 6A), indicating substantial suppression of ME49-induced inflammatory responses. IFN-γ levels were also significantly decreased in the GRA14 group (Figure 6B), although the magnitude of reduction was less pronounced than that observed for IL-6. Analysis of parasite burden revealed that brain cyst numbers were reduced by approximately 8.8-fold in GRA14-immunized mice relative to naive + challenge animals (Figure 6C). This substantial decline demonstrates highly effective suppression of cyst formation.

Moreover, mice in the GRA14 group maintained stable body weight throughout the 40-day observation period, with no signs of progressive wasting (Figure 6D). Importantly, survival remained at 100% through 40 dpi (Figure 6E). In contrast, unimmunized mice began to show progressive weight loss around 20 dpi, followed by rapid deterioration after 30 dpi. Survival declined to 66.7% by 35 dpi, and by 40 dpi, all mice exhibited >20% body weight loss or had succumbed to infection. These findings suggest that GRA14 VLP immunization significantly reduces neuroinflammation, suppresses brain cyst formation, and provides significant protection against lethal ME49 challenge.

### 3.7. GRA14 VLPs Offer Long-Term Humoral Immunity and Partial Protection Against Delayed Lethal ME49 Challenge

**Figure 7:**
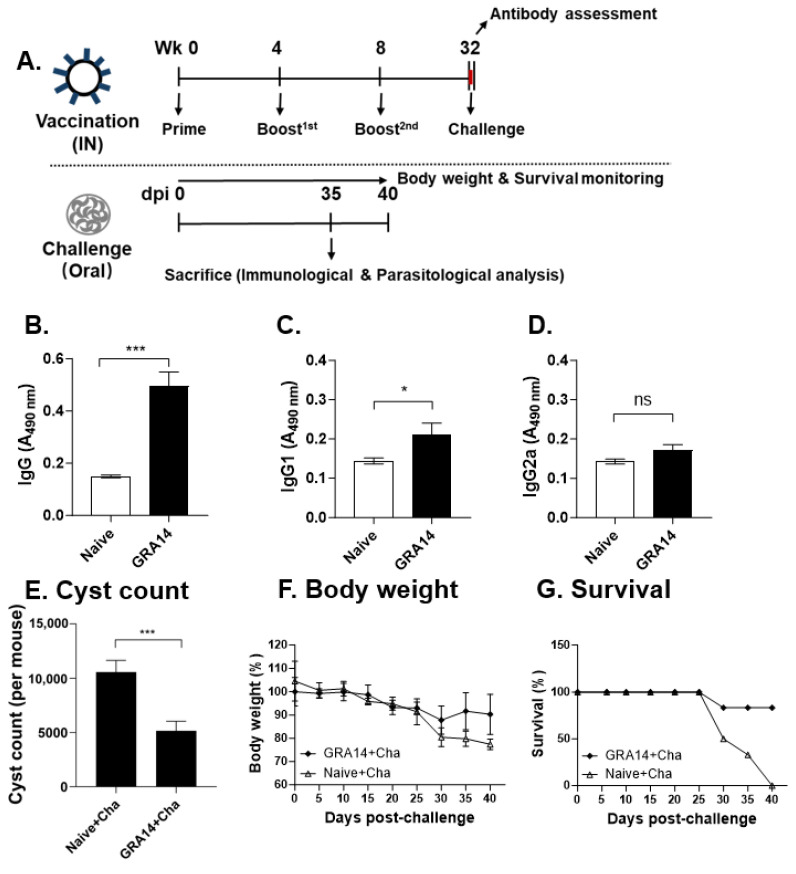
Long-lasting humoral immunity and protection efficacy against delayed lethal ME49 challenge. (**A**) Experimental timeline. Mice were immunized intranasally with GRA14 VLPs following the same three-dose schedule used in the short-term study and challenged with ME49 at 32 wpv (0 dpi). (**B**–**D**) Serum was collected for antibody analysis. (**E**) At 35 dpi, mice were sacrificed to collect brain tissues. Brain cyst burden was quantified by a microscope. (**F**) Bodyweight changes and (**G**) survival were monitored after delayed challenges until 40 dpi. Data are presented as mean ± SD. * *p* < 0.05, *** *p* < 0.001, ns, no significance. For survival analysis expressed as the percentage of surviving animals per group at each time point, SD is not applicable. Wk, week; VLPs, virus-like particles; IN, intranasal; dpi, days post-infection.

To determine whether GRA14 VLP immunization induces durable humoral immunity and long-term protective effects against a delayed ME49 infection, mice that received intranasal vaccinations identical to the short-term protocol were challenged at 32 wpv, followed by serum collection for antibody analysis. At 35 dpi, mice were sacrificed for assessment of brain cyst burden and clinical outcomes (Figure 7A). Serological analysis showed that GRA14-specific IgG titers remained elevated at 32 wpv, approximately two-fold higher than those in naive controls (Figure 7B), demonstrating persistence of long-term humoral immune responses. IgG subclass evaluation revealed a maintained increase in IgG1 (Figure 7C), whereas IgG2a levels did not differ significantly between the GRA14-immunized and the naive control (Figure 7D).

Assessment of chronic infection revealed that brain cyst numbers were reduced by approximately 2.5-fold in GRA14-immunized mice compared with naive + challenge animals (Figure 7E). However, the magnitude of reduction was lower than that observed in the short-term challenge study. Clinical outcomes further reflected partial yet meaningful long-term protection. Both the GRA14 and unimmunized groups exhibited bodyweight decline after infection. However, in the GRA14 group, only a small subset of mice dropped below 80% bodyweight at ~30 dpi, and most survivors showed gradual recovery thereafter (Figure 7F). Survival analysis revealed 83% survival at 40 dpi in the GRA14-immunized group (Figure 7G). In contrast, naive + challenge mice experienced sharp weight loss beginning at ~25 dpi, with 50% survival at 30 dpi, decreasing to 33% at 35 dpi, and 0% survival by 40 dpi, with all animals meeting the >20% weight-loss humane endpoint. Collectively, although protective efficacy is reduced compared with the short-term challenge model, GRA14 VLP immunization induces durable antibody responses detectable at 32 wpv and confers significant long-term protection against a delayed lethal ME49 challenge.

## 4. Discussion

In this study, we used an influenza-based VLP platform to present *T. gondii* GRA14 and assessed its immunogenicity and protective efficacy in short- and long-term lethal ME49 challenge models. GRA14 VLPs induced strong and durable antibody responses together with cellular immune activation, leading to reduced cerebral inflammation, lower brain cyst burden, and improved survival, with protection maintained even after a 6-month delayed challenge.

GRA14’s strong vaccine performance is likely linked to its biological role. As a dense granule protein positioned at the PV membrane and the whole intravacuolar network, GRA14 lies directly at the host–parasite interface, where it contributes to vacuole integrity and nutrient exchange, which are essential for chronic infection [12]. This exposed and functionally critical localization provides a strong rationale for targeting GRA14 as a vaccine antigen. Moreover, our bioinformatic analysis confirmed that GRA14 possesses a membrane topology in which the C terminus is oriented toward the host cell cytosol and the N-terminal luminal domain faces the PV interior, consistent with previous reports [12]. Such a configuration is particularly advantageous for anchoring on M1-based VLPs, enabling stable insertion into the VLP envelope, as verified by TEM and Western blot analysis in the present study. This structural compatibility provides the mechanistic basis for the immune activation and protective efficacy observed in this study.

In the humoral compartment, GRA14 VLPs induced markedly elevated antigen-specific IgG titers, reaching nearly tenfold higher levels than controls at 10 wpv and remaining approximately twofold higher at 32 wpv, indicating the establishment of durable serological memory. This long-term persistence aligns with observations from natural *T. gondii* infection, in which IgG titers in pregnant women can remain detectable for many years or even decades [27]. Sustained IgG responses are particularly important for controlling chronic *T. gondii* infection, as IgG contributes to limiting parasite dissemination through tachyzoite neutralization, inhibition of cyst reactivation, and Fc receptor–dependent elimination functions [28,29].

IgG1 and IgG2a subclass analyses showed that both isotypes were elevated following GRA14 VLP immunization, consistent with previous studies using calcium-phosphate nanoparticles encapsulating GRA14 [30], and indicating a relatively balanced Th1/Th2 response. This pattern aligned with cytokine production by splenocytes upon TLA stimulation in the present study, where IFN-γ was markedly increased, as expected for intracellular parasite control [31], while IL-4 and IL-5 were also elevated, suggesting concomitant activation of Th2-associated mucosal humoral responses [32]. Because excessive Th1 inflammation can also cause tissue damage [33], the balanced profile induced by GRA14 VLPs may help achieve effective parasite control while minimizing immunopathology. Notably, the durability of IgG subclasses diverged during long-term immunity. IgG1 remained significantly elevated at 32 wpv, whereas IgG2a declined to levels comparable to unimmunized controls. This shift suggests a temporal remodeling of Th1/Th2 immune memory toward a more antibody-dependent, Th2-leaning profile during chronic protection. Although many *T. gondii* vaccines, such as rGRA12, pVAX-ROP54, and pVAX1-MYR1 reported in previous studies, have been reported to induce mixed Th1/Th2 responses shortly after immunization (within two months) [34,35,36], long-term subclass dynamics remain insufficiently characterized. Our findings may therefore provide a useful reference for understanding the evolution of humoral immunity during extended protection.

The cellular basis for this sustained humoral response was further supported by increased IgG- and IgA-secreting ASCs in both spleen and bone marrow, indicating activation of short-lived plasma blasts as well as the establishment of long-lived plasma cells. The concurrent elevation of GC B cells and memory B cells, which are supported by CD4^+^ T cell activation, suggests that GRA14 VLPs effectively drive classical GC reactions, promoting affinity maturation and durable antibody production. This outcome is consistent with the inherent advantage of VLPs to present multivalent antigens in a virus-like geometry that enhances B-cell receptor crosslinking [37,38]. Beyond humoral immunity, we observed an increase in CD8^+^ T cells in the immunized group, indicating that GRA14 VLPs also enhance cytotoxic T-cell populations capable of producing effector cytokines and directly targeting infected cells. Although CD8^+^ T-cell activation is not unique to VLP-based immunization, while similar responses have been reported for GRA-containing DNA as well as SAG protein subunit vaccines [39,40], such activation remains essential for shaping robust adaptive immunity and contributing to effective parasite control.

In the short-term challenge model, GRA14 VLP vaccination led to an approximately 8.8-fold reduction in brain cyst burden, a level of parasite control consistent with the “low-cyst–low-inflammation” homeostasis observed in chronic toxoplasmosis immunity [41]. Compared with previously reported VLP vaccines incorporating other *T. gondii* antigens, such as ROP13 VLPs (~1-fold reduction), ROP4 VLPs (~3-fold reduction), or CST1 VLPs (~7-fold reduction) [22,26], GRA14 VLPs showed a comparatively stronger impact on cyst limitation. This enhanced efficacy may relate to GRA14’s more intimate association with PV organization and cyst development during chronic infection. Differences in immunization regimes may also contribute. The mucosa-targeting potential of intranasal vaccination, and the use of a three-dose regimen, whereas comparable studies employed only two immunizations, may collectively strengthen protective immunity by establishing a more robust immune barrier between the parasite entry site and its target tissues. These variables highlight the importance of both antigen selection and immunization strategies in shaping protective outcomes. Protection remained evident but diminished in the delayed-challenge model, where cyst numbers declined by ~2.5-fold and survival reached 83%. Although reduced compared with short-term outcomes, these findings suggest that residual antibodies and memory responses still contribute meaningfully to parasite control during long-term immunity.

Although GRA14 VLP vaccination conferred 100% protection against lethal chronic toxoplasmosis in the short-term challenge model, brain cysts were not completely eliminated. In addition, a clear decline in protective efficacy was observed in the long-term immunity group compared with the short-term challenge. To address this limitation, future work should therefore explore strategies to further enhance vaccine efficacy, including optimization of antigen dose, immunization intervals, adjuvant formulations, and combinatorial antigen approaches before being translated to other species. In particular, GRA14 is associated with parasitophorous vacuole membrane organization and chronic-stage parasite persistence, whereas other antigens target distinct biological processes such as host cell invasion, intracellular replication, or cyst formation (e.g., SAGs, ROPs, or cyst wall–associated proteins). Combining GRA14 with such antigens may therefore broaden immune recognition across multiple parasite compartments and life-cycle stages, potentially resulting in more effective suppression of residual cyst burden. Moreover, although we profiled major immune cell populations, the roles of specific B- and T-cell subsets in sustaining long-term protection remain unclear. Defining these memory compartments more precisely will be important for understanding the partial decline in immunity observed during delayed challenge.

## 5. Conclusions

In summary, our present study demonstrates that GRA14-displaying influenza VLPs elicit significant humoral and cellular immune responses and provide substantial protection against both short-term and delayed lethal chronic *T. gondii* challenge, highlighting the relevance of GRA14 as a potential vaccine candidate and underscoring the VLP platform as a promising strategy for next-generation toxoplasmosis vaccines.

## Data Availability

Data presented in this study are contained within the article. Further inquiries can be directed to the corresponding author.
